# Construction of lncRNA-related ceRNA regulatory network in diabetic subdermal endothelial cells

**DOI:** 10.1080/21655979.2021.1936892

**Published:** 2021-06-14

**Authors:** Jiangbo Wan, Bo Liu

**Affiliations:** a Department of Burns, The First Affiliated Hospital of Nanchang University, Nanchang, Jiangxi China; b Department of Burns and Surgery, The Fourth Affiliated Hospital of Guangxi Medical University, Liuzhou, Guangxi, China

**Keywords:** Long non-coding rnas, diabetic vasculopathy, endothelial cells

## Abstract

Long non-coding RNAs (lncRNAs) were considered to be involved in vascular complications in diabetes mellitus, but still only limited knowledge in this regard has been obtained. Herein, we further explored the roles of lncRNAs and mRNAs in diabetic vasculopathy (DV) through conducting bioinformatics analysis using data set downloaded from GEO database. The differentially expressed lncRNAs and mRNAs were identified by edge package. GO enrichment analysis and KEGG pathway analysis were performed based on clusterprofiler package. The relationship between lncRNA and miRNA was predicted using starBase database, and the potential mRNAs targeted by miRNAs were predicted by TargetScan, miRTarbase and miRDB database. The string database was used to analyze the protein-protein interaction (PPI). As a result, a total of 12 lncRNAs and 711 mRNAs were found to be differentially expressed in the diabetic subdermal endothelial cells compared with normal controls. A ceRNA network was established, which was composed of seven lncRNA nodes, 49 miRNA nodes, 58 mRNA nodes and 183 edges, and MSC-AS1 and LINC01550 may serve as key nodes. GO function enrichment analysis showed enrichments of epithelial cell proliferation, intercellular junction, and cell adhesion molecule binding. KEGG pathway analysis revealed 33 enriched pathways. PPI protein interaction analysis identified 57 potential ceRNA-related proteins. Overall, this study suggests that multiple lncRNAs, specifically MSC-AS1 and LINC01550, may play an important role in DV development and they are like to be developed as the therapeutic targets for DV. However, further experiments in vitro and in vivo should be conducted to validate our results.

## Graphical Abstract

**Figure:**
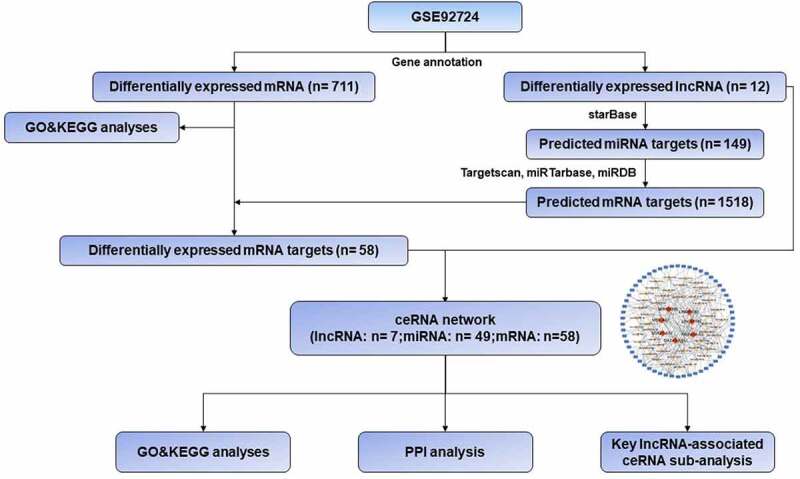

## Introduction

Diabetes mellitus is a worldwide disease with high incidence and morbidity, and it is characterized with chronic hyperglycemia caused by multiple causes. The pre-diabetes changes and diabetes mellitus (DM) have affected 425 million people worldwide in 2017, and it was predicted to rise to 629 million by 2045 [1,2]. The long-term increase of blood glucose can lead to the damage of large blood vessels and micro vessels and endanger the heart, brain, kidney, peripheral nerve, eyes and feet. According to the statistics of the World Health Organization, there are more than 100 kinds of complications [3,4]. Diabetic vasculopathy (DV) is one of the most common and severe complications of both type 1 and type 2 diabetes mellitus. DV refers to a series of vascular damages and is related to many other complications, such as diabetic retinopathy, diabetic nephropathy and diabetic foot ulcer (DFU). DV and its relevant complications have been considered to influence the prognosis of patients with DM, so it is necessary to fully elucidate its mechanisms, so as to develop strategies to prevent or deal with it.

The damage of vascular endothelial cell is the most crucial step during DV development. Under physiological condition, insulin can interact with its receptor on endothelial cells to activate eNOS, upregulates NO release and reduces ET-1 secretion, consequently inducing the vasodilation of blood vessels [5]. However, the insulin and glucose uptake by endothelial cells decrease and even insulin resistance occurs ultimately when endothelial cells are damaged [6]. Additionally, oxidative stress has also been proposed to promote DV through overproduction of reactive oxygen species (ROS), which can induce endothelial cell apoptosis by negatively regulating Akt/NF-kappaB pathway [7]. Furthermore, evidence exists that mitochondrial energy metabolism dysfunction and advanced glycation end products (AGEs) participate in DV development as well, since they are able to disturb the physiological stability of endothelial cells, thereby inducing endothelial cell death [8,9]. To date, our knowledge of the damage of vascular endothelial cells in DM is still insufficient, and much effort should be paid to investigate its pathogenesis and further determine its roles in DV development.

Long non-coding RNA (LncRNA) is a class of noncoding RNA molecules with longer than 200 nucleotides that lack protein-coding functions. Recently, increasing studies suggested that multiple lncRNAs are dysregulated in DM and involved in the pathogenesis of DM. For instance, Ruan et al. revealed that lncRNA-p3134 contributes to glucose metabolism and insulin signal transduction in pancreatic B cells [10]. Feng et al. demonstrated that lncRNAs modulate inflammation in diabetic peripheral neuropathy as ceRNAs targeting miR-146a-5p [11]. Kong et al. reported that lncRNA LEGLTBC regulates glucolipotoxicity-induced INS-1 beta cell oxidative stress and apoptosis via the miR-34a/SIRT1 axis [12]. Nevertheless, there are few studies exploring the role of lncRNAs in DV.

To develop new strategies for preventing and treating DV, in this study, we tried to investigate the role of lncRNAs and their potential mechanisms in DV by the bioinformatics analysis using a high throughput sequencing dataset (GSE92724) and miRNA-predicted websites. As a result, we identified 12 differentially expressed lncRNAs in the diabetic subdermal endothelial cells. Then, the ceRNA regulatory network was established based on those lncRNAs and two key ceRNA sub-networks may be involved in the pathogenesis of DV. Overall, this study helps us to better understand the effects of lncRNAs in the dermal endothelial cells whose damage is involved in the pathogenesis of DV.

## Method

### Data acquisition

The expression of dermal endothelial cells of type 2 diabetic patients processed by high throughput sequencing was downloaded from the Gene Expression Omnibus (GEO) database (https://www.ncbi.nlm.nih.gov/geo/). The GSE92724 dataset included 4 diabetic patients and 6 control individuals. The mRNAs and lncRNAs were distinguished by using the annotation information obtained from Genecode database. Totally, 18,206 mRNAs and 2025 lncRNAs were obtained from the high throughput sequencing dataset.

### Functional enrichment analysis of differentially expressed mRNAs and lnRNAs

The differentially expressed mRNAs and lnRNAs of dermal blood endothelial cell between diabetic patients and the healthy controls were screened using ‘edgeR’ [13] package in the R software (version 3.6.1), based on the cutoff values: |logFC| > 1 and the false discovery rate (FDR) < 0.05. The R package ‘clusterProfiler’ [14] was used to implement the functional annotation of Gene ontology (GO) enrichment analysis and Kyoto Encyclopedia of Genes and Genomes (KEGG) pathway analysis of the differentially expressed ARGs. Adjusted P < 0.05 was considered statistically significant.

### Construction of ceRNA network

The subcellular localizations of lncRNAs were predicted in the lncLocator database by uploading the FASTA sequences downloaded from GenBank database (https://www.ncbi.nlm.nih.gov/) [15]. The DElncRNA-miRNA interactions were predicted by using the starBase database (http://starbase.sysu.edu.cn/) [16]. The predicted targeted miRNAs were used to forecast the potential interactions with mRNAs by using the Targetscan (http://www.targetscan.org/vert_72/) [17], miRTarbase (http://mirtarbase.cuhk.edu.cn/php/index.php) [18] and miRDB (http://mirdb.org/) [19] database. After overlapping the predicted mRNAs and the DEmRNAs, the regulated Visualization of the lncRNA-miRNA-mRNA ceRNA network was constructed using Cytoscape software (Version 3.7.2) [20]. In the ceRNA network, biological molecules were presented as nodes, while node-node interactions were presented as edges. Red diamonds, yellow triangles and blue rectangles in the ceRNA network indicated lncRNAs, miRNAs, and mRNAs, respectively. lncRNA-miRNA-mRNA interactions were presented as gray edges.

### Protein-Protein Interaction (PPI) network analysis of mRNAs in ceRNA network

The PPI network of mRNAs involved in the ceRNA network was established through an online search tool, Search Tool for the Retrieval of Interacting Genes (STRING) database (https://string-db.org) with a confidence score > 0.40 [21].

### Conduction of Key ceRNA subnetwork

Network degree of each node in the ceRNA network was evaluated in Cytoscape. The lncRNA with node degree ≥6 was considered as a hub lncRNA. Then, the hub lncRNA-associated sub-networks were established by using the information between the relationship of hub lncRNA-miRNA and the relationship of miRNA-mRNA.

## Result

### Identification of differentially expressed mRNAs and lnRNAs

**Figure 1.**
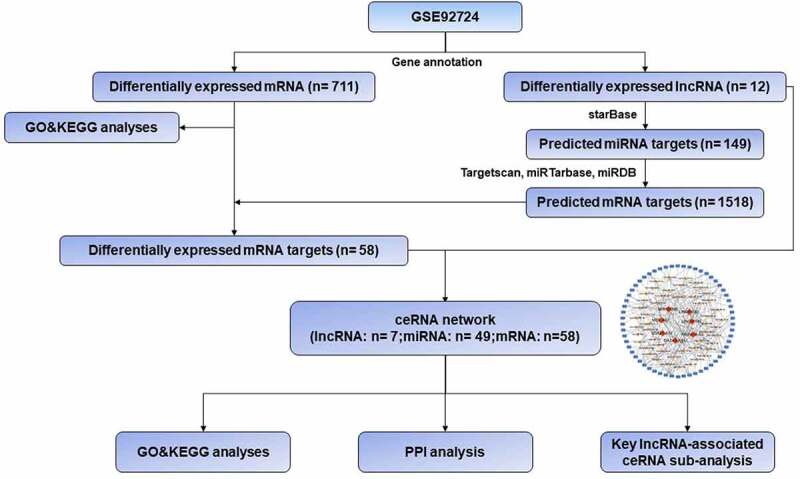
Workflow of the present study

**Figure 2.**
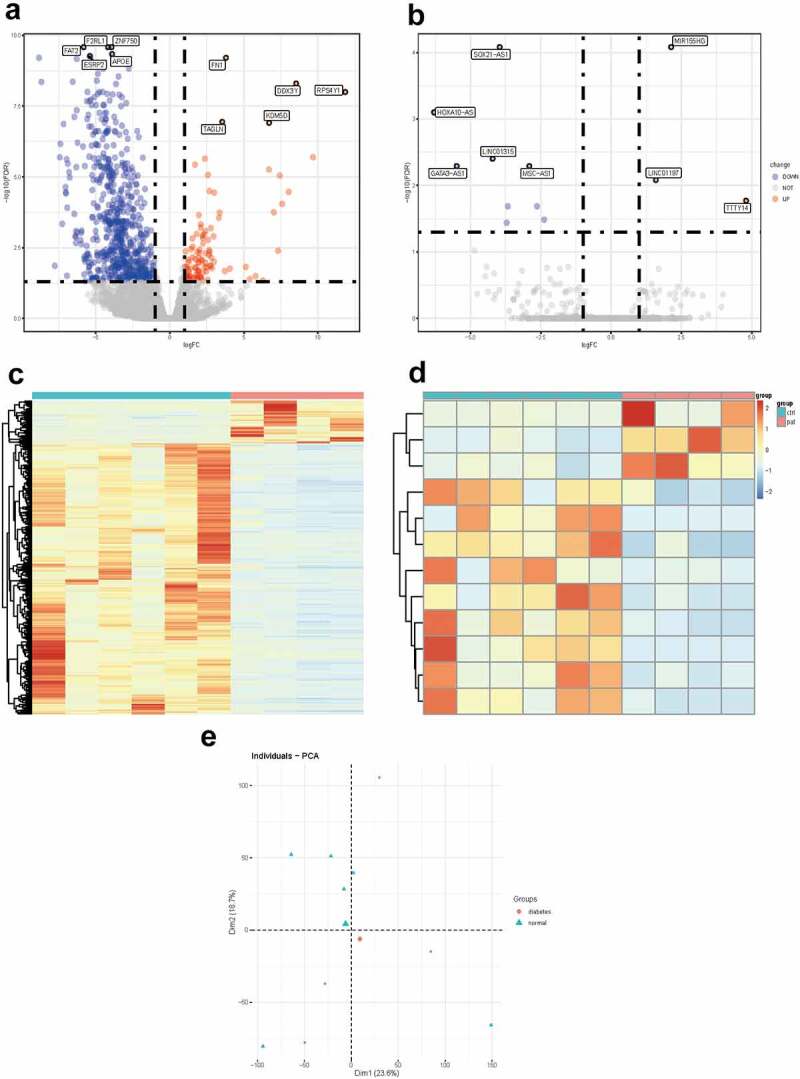
**Differentially expressed mRNAs and lnRNAs in GSE92724**. (a) the volcano plot of the differentially expressed mRNAs. (b) the volcano plot of the differentially expressed lncRNAs. (c) the heatmap of the differentially expressed mRNAs. (d) the heatmap of the differentially expressed lncRNAs. the red dots indicated up-regulated genes and the blue dots indicated down-regulated ones. the gray dots indicated genes which are not differentially expressed

In order to facilitate the understanding of the research, the entire workflow is presented in Figure 1. Raw data were downloaded from GSE92724 dataset in GEO database and 8206 mRNAs and 2025 lncRNAs were identified by using the annotation information downloaded from Genecode database. The edgeR package was performed to identify differentially expressed mRNAs and lncRNAs with a strict cutoff threshold of |logFC | > 1 and FDR <0.05. Compared with normal samples, 12 DElncRNAs and 711 DEmRNAs were differentially expressed, among which 9 lncRNAs and 614 mRNAs were downregulated as well as 3 lncRNAs and 97 mRNAs were upregulated in diabetic dermal blood endothelial cells (Table 1 and Supplementary Table S1). The volcano plots, heat maps and PCA plot of DElncRNAs and mRNAs of dermal blood endothelial cell between diabetic patients and the healthy controls are shown in Figure 2.

**Table 1.** The differentially expressed lncRNAs in GSE92724

LncRNA	Expression pattern	LogFC	FDR
MIR155HG	UP	2.11532	8.30E-05
SOX21-AS1	DOWN	−3.97818	8.30E-05
HOXA10-AS	DOWN	−6.31366	7.94E-04
LINC01315	DOWN	−4.23191	3.95E-03
GATA3-AS1	DOWN	−5.50971	5.11E-03
MSC-AS1	DOWN	−2.92726	5.11E-03
LINC01197	UP	1.57444	8.25E-03
TTTY14	UP	4.78030	1.70E-02
LINC00640	DOWN	−3.67468	2.06E-02
FAM182B	DOWN	−2.63573	2.06E-02
LINC01550	DOWN	−2.38106	3.26E-02
LINC01068	DOWN	−3.71202	3.63E-02

### Functional enrichment analysis of DEmRNAs

**Figure 3.**
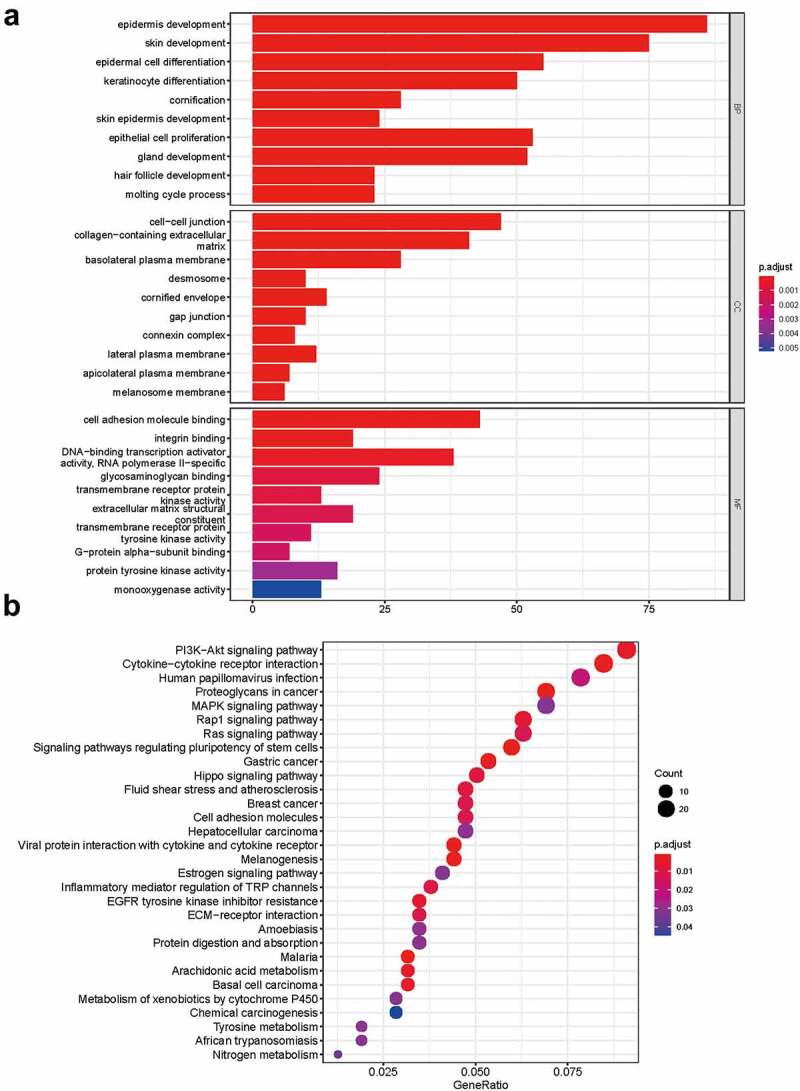
**Functional enrichment analysis of DEmRNA**. (a) GO enrichment analysis. (b) KEGG pathway analysis

To determine the functions of the significantly upregulated and downregulated DEmRNAs, GO enrichment analysis and KEGG pathway analysis were conducted by using ‘clusterProfiler’ R package. The BP analysis showed that the DEmRNAs were mostly enriched in epithelial cell proliferation, regulation of epithelial cell proliferation, and epithelial cell development. The CC analysis showed that the DEmRNAs were significantly enriched in cell-cell junction, collagen−containing extracellular matrix, and basolateral plasma membrane. The MF analysis showed that the DEmRNAs were significantly enriched in cell adhesion molecule binding, integrin binding and glycosaminoglycan binding. The KEGG pathways enriched analysis results showed that major pathways, including the PI3K−Akt signaling pathway, Cytokine−cytokine receptor interaction, MAPK signaling pathway and MAPK signaling pathway, were involved in the DEmRNAs. The results of the GO and KEGG functional enrichment analyses are presented in Figure 3. The complete list of the GO and KEGG analyses is presented in Supplementary Table S2-S3.

### Construction of ceRNA network

**Figure 4.**
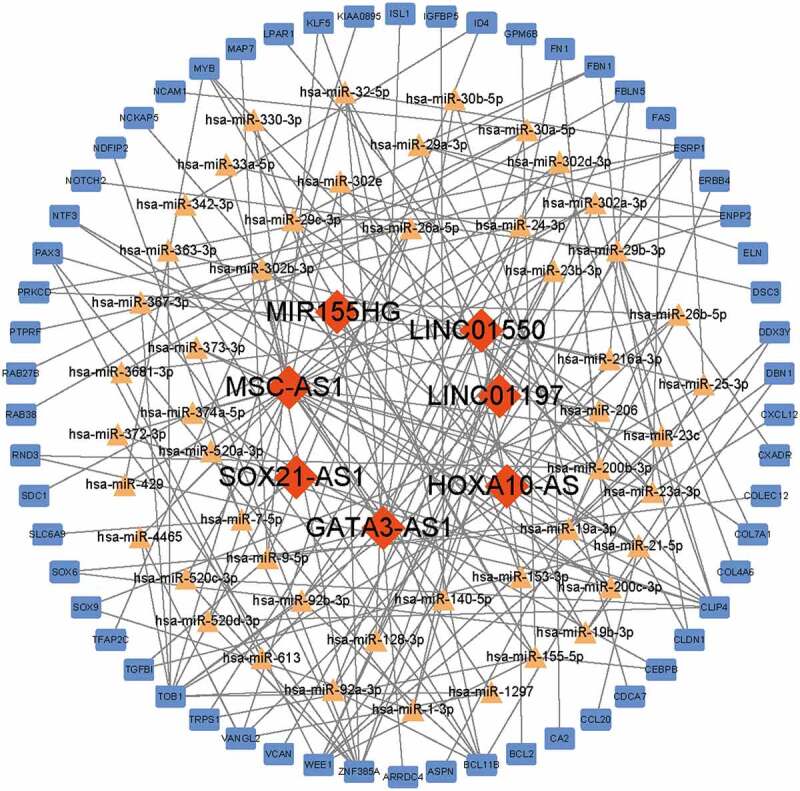
**Construction of ceRNA network**. Red diamonds indicated lncRNAs, yellow triangles indicated miRNAs and blue rectangles indicated mRNAs. Gray edges indicated the lncRNA-miRNA-mRNA interactions

To establish the ceRNA network, the DElncRNAs available in the starBase database were further analyzed and the subcellular localizations of these lncRNAs were predicted in the lncLocator database. All of the 7 DElncRNAs predicted were located outside the nuclear (Table 2). The potential DElncRNA targeted miRNAs were predicted using starBase database. Then, the DEmRNA-miRNA pairs were evaluated by the Targetscan, miRTarbase, and miRDB databases. When the DElncRNA-miRNA pairs and the DEmRNA-miRNA pairs contained a common miRNA, they were chosen to conduct the ceRNA network. In total, the ceRNA network was conducted with 183 edges and 114 nodes, including 7 lncRNAs, 49 miRNAs and 58 mRNAs (Figure 4).

**Table 2.** Subcellular localization of DElncRNAs predicted in the lncLocator database

LncRNA	Cytoplasm	Nucleus	Ribosome	Cytosol	Exosome	Location
MIR155HG	0.667308236	0.245784716	0.022277375	0.037860786	0.026768887	Cytoplasm
SOX21-AS1	0.095079593	0.168018316	0.119419437	0.522095575	0.095387079	Cytosol
GATA3-AS1	0.051540579	0.01097351	0.472667371	0.31724024	0.1475783	Ribosome
HOXA10-AS	0.001447872	0.000274422	0.995587178	0.002264769	0.000425759	Ribosome
MSC-AS1	0.884901537	0.04950467	0.017267884	0.040407388	0.007918522	Cytoplasm
LINC01197	0.393279828	0.321648641	0.021892264	0.038890803	0.224288464	Cytoplasm
LINC01550	0.87973863	0.03967279	0.016868801	0.061817511	0.001902268	Cytoplasm

### Functional enrichment analysis of the ceRNA Network

**Figure 5.**
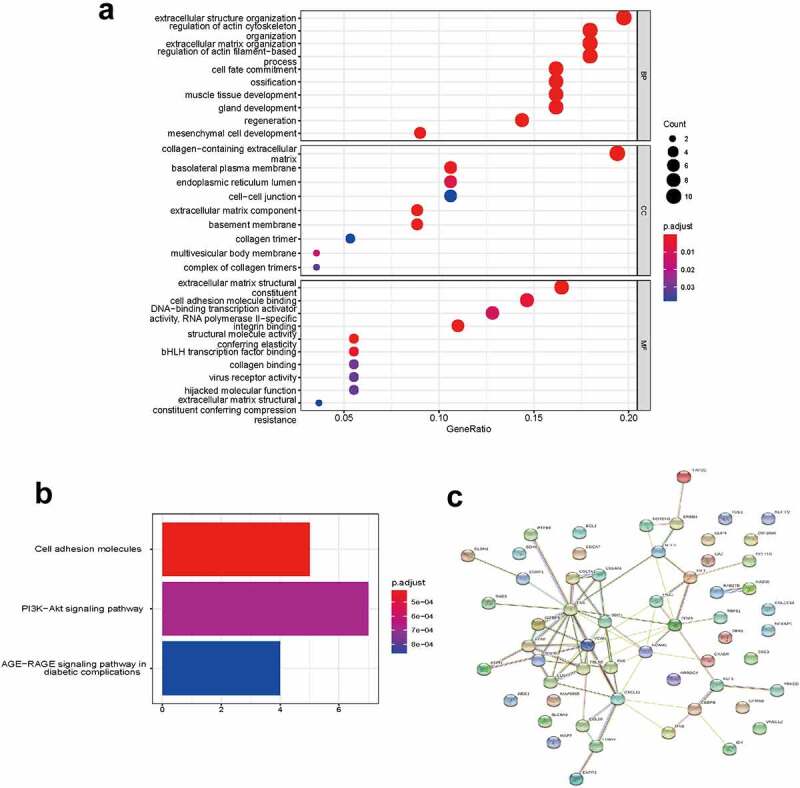
**Functional enrichment analysis of the ceRNA network**. (a) GO enrichment analysis. (b) KEGG pathway analysis. (c) PPI analysis. Edges indicated the protein-protein interactions

To gain further insight into the functions of the targeted mRNAs in ceRNA network, GO enrichment analysis and KEGG pathway analysis were conducted (Figure 5). The complete list of the GO and KEGG analyses is presented in Supplementary Tables S4-S5. The BP analysis showed that the targeted mRNAs were mainly enriched in extracellular structure organization, regulation of actin cytoskeleton organization, and extracellular matrix organization. The CC analysis showed that the targeted mRNAs were mainly enriched in collagen−containing extracellular matrix, basolateral plasma membrane, and basolateral plasma membrane. The MF analysis showed that the targeted mRNAs were mainly enriched in extracellular matrix structural constituent, integrin binding and cell adhesion molecule binding. The KEGG pathway enriched analysis results showed that the targeted mRNAs were mainly enriched in cell adhesion molecules, PI3K-Akt signaling pathway and AGE−RAGE signaling pathway in diabetic complications. The PPI network of mRNAs in the ceRNA network generated by the 57 nodes and 69 edges is shown in Figure 5.

### Conduction of Key ceRNA subnetwork

**Figure 6.**
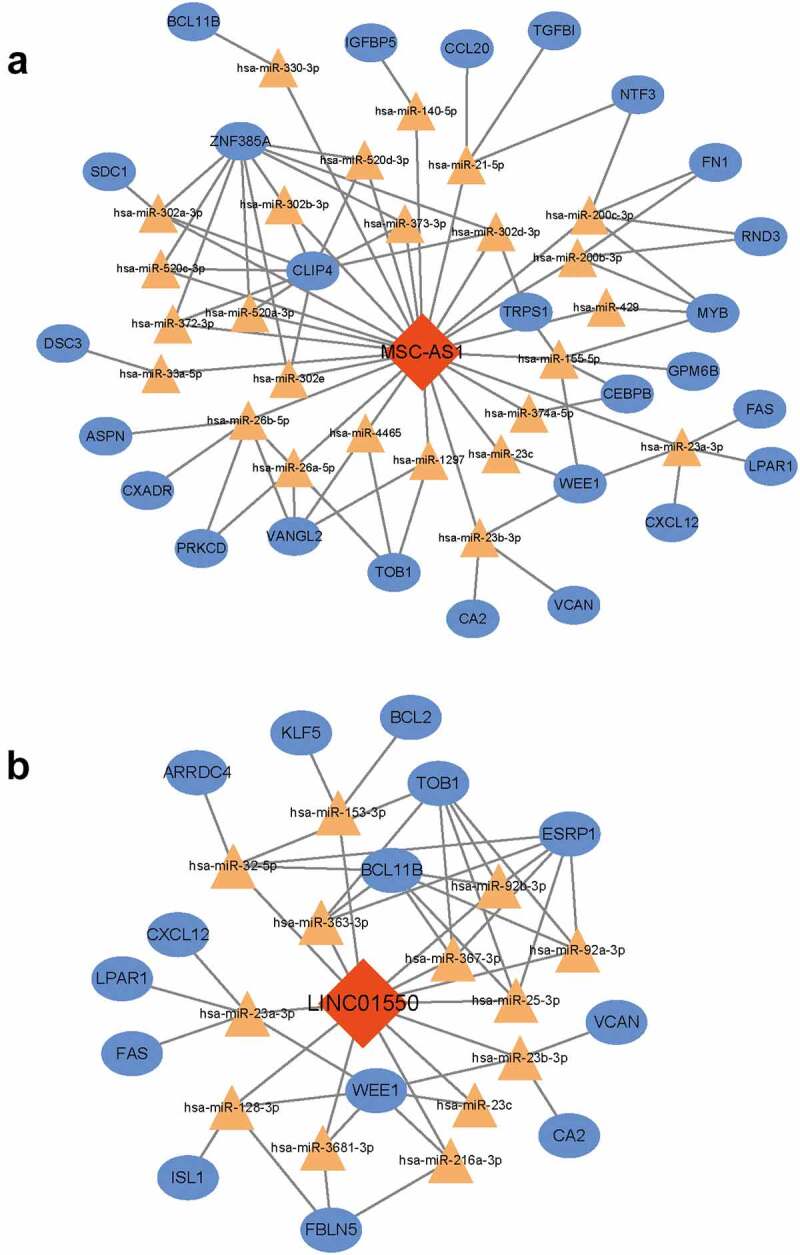
**Conduction of key ceRNA sub-network**. (a) MSC-AS1-associated ceRNA sub-network. (b) the LINC01550-associated ceRNA sub-network. Red diamonds indicated lncRNAs, yellow triangles indicated miRNAs and blue rectangles indicated mRNAs. Gray edges indicated the lncRNA-miRNA-mRNA interactions

To figure out the key lncRNA-associated ceRNA subnetwork, the node degree of each gene in this ceRNA network was evaluated by using a built-in ‘cytoHubba’ tool of Cytoscape. As listed in Table 3, a total of 14 genes with node degree no less than six were disclosed, including 2 lncRNAs (MSC-AS1, LINC01550). Then, the interactions of hub lncRNAs were extracted to conduct the related ceRNA subnetworks. As shown in Figure 6, the MSC-AS1-associated ceRNA sub-network was composed of 25 miRNAs, 26 mRNAs and 85 edges. The LINC01550-associated ceRNA sub-network was composed of 13 miRNAs, 14 mRNAs and 49 edges.

**Table 3.** The differentially expressed genes in ceRNA (node degree ≥ 6)

Gene	Gene type	Node degree
MSC-AS1	lncRNA	25
LINC01550	lncRNA	13
TOB1	mRNA	10
CLIP4	mRNA	9
ZNF385A	mRNA	9
hsa-miR-155-5p	miRNA	7
hsa-miR-29b-3p	miRNA	7
WEE1	mRNA	7
BCL11B	mRNA	7
hsa-miR-23a-3p	miRNA	6
hsa-miR-26b-5p	miRNA	6
hsa-miR-29 c-3p	miRNA	6
hsa-miR-9-5p	miRNA	6
ESRP1	mRNA	6

## Discussion

Diabetic vasculopathy is a defect in blood vessels caused by hyperglycemia, which causes severe organ-specific complications, such as nephropathy, retinopathy, myocardial infarction and cerebrovascular disease [22,23]. Evidence shows that the damage of vascular endothelial cells plays a crucial role in the occurrence and development of DV. Thus, to comprehensively understand the molecular mechanisms for the damage of vascular endothelial cells may help to find new strategies for the prevention and therapy of DV. In recent years, lncRNAs were found to regulate vascular endothelial cell functions, indicating their potential roles in DV development [24]. For example, lncRNA-MIAT can regulate microvascular dysfunction by functioning as a ceRNA [25]. In addition, Gong et al. suggested that LncRNA TDRG1 aggravates retinal microvascular endothelial cell dysfunction in diabetic retinopathy through upregulating VEGF [26]. With the development of high-throughput sequencing, a large number of novel lncRNA were identified in DV. However, the association of these lncRNAs with DV development remains largely unknown.

In this study, we tried to further identify lncRNAs involved in DV and preliminarily explore the potential mechanisms by conducting the bioinformatics analysis of publica dataset (GSE92724). A total of 12 differently expressed lncRNAs in subdermal endothelial cells were identified between 4 diabetic patients and 6 control individuals. Then, a ceRNA network was finally established with 183 edges and 114 nodes, including 7 lncRNAs, 49 miRNAs and 58 mRNAs. Additionally, KEGG pathway analysis further revealed that 33 signaling pathways were enriched, such as PI3K/Akt pathway, Rap1 pathway and MAPK pathway. Strikingly, previous studies showed that those pathways are closely associated with DV development. Lu et al. suggested that PI3K/AKT axis meditated the function of miR-21 in promoting retinal vascular endothelial cell viability and angiogenesis in diabetic retinopathy [27]. Wilson et al. revealed that the Rap1-Rasip1 signaling transduction plays a critical role in stabilizing and remodeling vascular endothelials besides junction [28]. Evidence indicates that aberrant activation of MAPK-JNK pathway delays wound healing in diabetic foot patients by aggravating inflammation. It has been revealed that extracellular matrix protein synthesis and capillary basement membrane thickening in microvasculatures can significantly contribute to diabetic microangiopathy [29]. Consistently, by GO analysis, we found that mRNAs in the ceRNA network were mainly enriched in extracellular structure organization, collagen-containing extracellular matrix and extracellular matrix structural constituent.

Among seven differentially expressed lncRNAs, MSC-AS1 and LINC01550 that were markedly downregulated in diabetic subdermal endothelial cells were found to serve as key nodes. To date, no studies were performed to explore the roles of MSC-AS1 and LINC01550 in DM. However, numerous recent studies suggested that MSC-AS1 is dysregulated in multiple types of cancers and performs an oncogenic function in tumor progression [30,31], whereas LINC01550 was rarely studied. As mentioned above, our KEGG pathway analysis suggested that the differentially expressed lncRNAs may regulate DV development via the PI3K/Akt pathway. Coincidentally, dysregulation of MSC-AS1 expression was found to promote tumor progression in glioma and osteosarcoma through the PI3K-Akt pathway [32,33], which may further support the important role of MSC-AS1/PI3K-Akt pathway in DV to a certain extent.

There are some limitations in this study, and it should be seriously considered when interpreting our results. To begin with, the conclusion of this study was merely made based on the bioinformatic analysis. Therefore, in future, necessary experiments in vitro and in vivo should be performed to further validate our findings. Additionally, the publica data we used only included 4 diabetic patients and 6 control individuals, so the limitation of small sample size may lead to overestimated conclusion. Hence, large sample and multi-center clinical studies should be conducted to confirm our results.

## Conclusion

In summary, 12 lncRNAs and 711 mRNAs were identified to differentially express in the diabetic subdermal endothelial cells versus normal controls. Based on these lncRNAs and mRNAs, a lncRNA-miRNA-mRNA interaction network was established, which was composed of seven lncRNA nodes, 49 miRNA nodes, 58 mRNA nodes and 183 edges, and MSC-AS1 and LINC01550 were found to serve as key nodes in the network. Overall, this study suggested that multiple lncRNAs, specifically MSC-AS1 and LINC01550, may play an important role in DV development and they are like to be developed as the therapeutic targets for DV. However, further experiments in vitro and in vivo should be conducted to validate our results.

## Supplementary Material

Supplemental Material
