# Supplementary material for: Construction of lncRNA-related ceRNA regulatory network in diabetic subdermal endothelial cells
Source: Bioengineered. 2021 Jun 14;12(1):2592–602. doi: 10.1080/21655979.2021.1936892 (PMC8806614; doi:10.1080/21655979.2021.1936892)
Supplement: Supplemental Material [file KBIE_A_1936892_SM5521.zip › Supplementary Table caption.docx]

**Supplementary Table 1. The differentially expressed mRNAs in GSE92724.**

**Supplementary Table 2. GO enrichment analysis of DEmRNAs.**

**Supplementary Table 3. KEGG enrichment analysis of DEmRNAs.**

**Supplementary Table 4. GO enrichment analysis of mRNAs in the ceRNA.**

**Supplementary Table 5. KEGG enrichment analysis of mRNAs in the ceRNA.**
